# A Non-Randomized Comparison of Online and In-Person Formats of the Canadian Androgen Deprivation Therapy Educational Program: Impacts on Side Effects, Bother, and Self-Efficacy

**DOI:** 10.3390/curroncol31090373

**Published:** 2024-08-28

**Authors:** Lauren M. Walker, Carly S. Sears, Erik Wibowo, John W. Robinson, Andrew G. Matthew, Deborah L. McLeod, Richard J. Wassersug

**Affiliations:** 1 Division of Psychosocial Oncology, Department of Oncology, University of Calgary, Calgary, AB T2N 4N1, Canada; carly.sears@ucalgary.ca (C.S.S.);; 2 Arnie Charbonneau Cancer Institute, University of Calgary, Calgary, AB T2N 4N1, Canada; 3 School of Medical Sciences, Faculty of Medicine and Health, University of Sydney, Sydney 2006, Australia; 4 Division of Urology, Department of Surgery, University of Toronto, Toronto, ON M5T 2SB, Canada; 5 School of Nursing, Faculty of Health, Dalhousie University, Halifax, NS B3H 4R2, Canada; dmcleod@carepath.ca; 6 Cellular & Physiological Sciences, Faculty of Medicine, University of British Columbia, Vancouver, BC V6T 2A1, Canada; richard.wassersug@ubc.ca

**Keywords:** androgen deprivation therapy, prostate cancer, patient education, side effect management, self-efficacy, psychosocial oncology

## Abstract

Although Androgen Deprivation Therapy (ADT) is effective in controlling prostate cancer (PCa) and increasing survival, it is associated with a myriad of side effects that cause significant morbidity. Previous research has shown that PCa patients starting on ADT are neither fully informed nor well-equipped to manage the breadth of ADT’s side effects. The ADT Educational Program (a 1.5 h interactive class plus a book) was developed as an evidence-based resource for patients dealing with ADT. Our aim here was to compare the efficacy of an online version of the class with a previously assessed in-person version of the class. Using mixed MANOVAs within a non-randomized comparison design, we assessed: (1) changes in patients’ experiences of self-efficacy to manage and bother associated with side effects approximately 10 weeks after attending a class, and (2) potential differences in these variables between online and in-person class formats. Side effect bother decreased from pre- to post-class but did not differ between in-person (*n* = 94) and online (*n* = 137) class cohorts. While self-efficacy to manage side effects was slightly higher post-class in both cohorts, the increase was not statistically significant. Average self-efficacy ratings were significantly higher among in-person versus online class participants (*p* < 0.05; η_p_^2^ = 0.128). Both online and in-person classes are associated with a significant reduction in the severity of side effect bother reported by PCa patients, suggesting non-inferiority of online versus in-person formats. Online classes offer greater accessibility to the program for patients outside the reach of in-person classes, increasing the availability of the program to more PCa patients and family members across Canada.

## 1. Introduction

Androgen Deprivation Therapy (ADT) is the mainstay treatment for locally advanced or de novo metastatic prostate cancer (PCa), and it is commonly used as neoadjuvant or adjuvant therapy to radiotherapy for the treatment of localized disease [1,2,3]. ADT reduces testosterone to castrate levels and is most commonly achieved chemically via gonadotropin-releasing hormone (GnRH) agonists and antagonists [1] or with androgen receptor pathway inhibitors [4]. Approximately half of all PCa patients are treated with ADT at some point during their cancer journey.

Although ADT is effective in managing PCa and increasing survival, androgen suppression has adverse effects that frequently lead to substantial morbidity [4,5]. These include increased risks of cardiovascular disease, diabetes type II, and metabolic syndrome; changes in body composition (e.g., increased percentage of fat versus muscle mass); decreased bone mineral density; hot flashes; breast growth and sensitivity; changes in cognitive function; depression; fatigue; sexual dysfunction (e.g., loss of libido and erectile dysfunction); and overall decreased quality of life across various domains [4,5,6,7].

Increasingly, men are prescribed an androgen receptor pathway inhibitor (ARPI) in addition to traditional ADT with LHRH agonists or antagonists [8]. Combination treatments with ARPIs are associated with improved survival, but these treatments tend to cause increased and more adverse side effects.

There is little agreement among uro-oncologists who start patients on ADT about what side effects men need to be aware of and prepared to manage [9,10]. Patients starting on ADT are often poorly informed and therefore ill-equipped to manage ADT side effects [11]. This was a key stimulus for the development of the ADT Educational Program (the ADT program) as an evidence-based educational resource for patients starting on ADT and their loved ones [12].

The ADT Program involves a 1.5 h interactive patient education session (herein referred to as the ADT class) and an accompanying book, *Androgen Deprivation Therapy: An Essential Guide for Men with Prostate Cancer and their Loved Ones*, now in its third edition (herein referred to as the ADT book) [13]. The ADT class and book together provide a comprehensive, patient-centered guide, with information about why ADT is prescribed, its mechanisms for cancer control, side effects, and evidence-based strategies for mitigating adverse effects [12]. The ADT program is based on principles of health behavior change to increase patients’ sense of self-efficacy in implementing management strategies and overcoming barriers to enacting health-promoting behaviors (e.g., increased physical activity) [12,14,15].

The initial launch of the ADT program exclusively involved in-person classes, offered at five pilot sites across Canada. An assessment of in-person classes found improvements in patient-reported self-efficacy to manage side effects of ADT [12].

In early 2018, following the successful implementation of in-person classes, the ADT program was adapted to an online format to increase accessibility. Of importance, the structure of the ADT class was maintained for the online version, and all participants continued to be offered a copy of the ADT book. Classes are held monthly as live, interactive, small-group sessions of up to 12 patients (plus loved ones), facilitated by PhD-level experts in the field.

Online patient education and supportive care interventions have increasingly been shown to benefit cancer patients [16]. However, studies generally involve heterogeneous cancer populations. There remains a dearth of research involving online education interventions for men on ADT, particularly in terms of side effect self-management [17,18,19]. According to a 2019 systematic review by Forbes and colleagues, preliminary findings suggest that online supportive care interventions for PCa patients are feasible, generally well-accepted by patients, and effective, though more rigorous studies are still needed [16]. Notably, only two of the 10 RCTs assessed by Forbes et al. compared online to in-person offerings [16]. The authors of a 2018 systematic review reached similar conclusions, noting that digital health interventions offer an effective means to improve self-management for cancer patients coping with treatment side effects (though not specific to prostate cancer) [17].

The online version of the ADT class commenced shortly before COVID-19 shut-downs of most in-person supportive care offerings. The online program continued throughout the pandemic, when people generally developed a sense of normalcy around online classes and sources of support. Given this background, we felt it important to compare the effectiveness of our educational resource in the online versus in-person format for men with PCa who had been prescribed ADT.

Our previous assessment of the in-person ADT program provided us with data to assess the online offering and to compare it to the in-person format [12]. Data pertaining to in-person classes were collected from 2014 to 2017 (prior to COVID-related closure of in-person classes). Data for online classes were collected from 2019 through 2021.

To remain consistent with the assessment of in-person classes, the authors chose to focus on self-efficacy to manage ADT side effects and severity of bother associated with side effect experiences as primary outcomes. Importantly, these variables reflect the foundational principles of the ADT Educational Program (i.e., to provide participants with education and evidence-based behavior-change tools to improve their sense of self-efficacy to manage ADT side effects and to reduce side effect-related bother) [12]. Moreover, self-efficacy has been shown to be an important outcome in studies involving lifestyle intervention and side effect management [20,21]. Based on findings from the assessment of the in-person ADT program, we also examined changes in self-reported occurrence of a range of ADT side effects prior to and approximately 10 weeks after attending the ADT class.

Accordingly, the objectives of the current study are to:

1. assess the effectiveness of the online ADT Educational Program in terms of pre-post changes in self-reported (i) side effect occurrence, (ii) bother associated with side effect experiences, and (iii) sense of self-efficacy to manage side effects;
2. compare the effectiveness of the online versus in-person ADT program format based on pre-post changes in participants’ self-reported side effect occurrence, bother, and management self-efficacy;
3. examine possible predictors of change in participants’ self-reported side-effect occurrence, bother, and management self-efficacy.

We predicted that participants in the online ADT program would report improvements in self-efficacy to manage ADT side effects and in bother severity by the 10-week follow-up. Since a proportion of participants were ADT-naïve at the time of attending the ADT class, we predicted that self-reported side effect occurrence would increase by the 10-week follow-up.

In terms of Objectives 2 and 3, we anticipated no significant differences between the online and in-person class formats. Given the known impacts of age on severity of ADT side effects (i.e., more severe side effects among younger men) [22], the authors anticipated that age would be predictive of changes in occurrence of, and bother associated with, ADT side effects.

## 2. Materials and Methods

### 2.1. Participants and Study Design

In-person ADT program participants were PCa patients who attended an in-person ADT class in one of five Canadian cities: Vancouver, Victoria, Calgary, Toronto, and Halifax. Online ADT program participants were PCa patients who attended the ADT class online and resided in Canada.

To be eligible, participants had to be fluent in English and planned to be on ADT for at least 6 months. Exclusion criteria included: (1) presence of symptomatic metastatic disease, (2) planned ADT duration of less than 6 months, (3) any psychological, familial, sociological, or geographic conditions that would impede ability to participate (i.e., to attend the ADT class and to complete both pre- and post-class questionnaires), (4) residing outside of Canada.

This study employed a non-randomized comparison design. Data pertaining to in-person class participants were collected prior to the availability of an online version of the class. Convenience sampling was used for both in-person and online class evaluations. Eligibility was determined during initial phone calls or email communication with the project assistant. While registering for a class (whether in-person or online format), participants were invited to participate in the optional “project evaluation study”. Attendance at an in-person or online class was not dependent on participation in the study.

### 2.2. Intervention

The ADT Educational Program was developed to provide PCa patients and loved ones with education about the side effects of ADT and evidence-based management strategies. Examples of management strategies include physical activity to manage fatigue, vitamin D and calcium to preserve bone health, and erectile aids to manage erectile dysfunction [6]. Participation in the program was predicated on attending the one-time, 1.5 h ADT class and reading the accompanying ADT book. Importantly, the program integrates health behavior change principles with patient education to support patients and their partners/loved ones in implementing side effect management strategies. Classes start with a didactic portion, during which participants are provided with information about ADT drugs, side effects, and evidence-based strategies that can help men manage side effects. This is followed by an interactive portion to help participants make lifestyle changes that support their wellbeing while on ADT. Here the class facilitators use established, evidence-based health behavior change strategies. Participants are invited to identify specific, actionable goals toward management of side effects (e.g., increase physical activity to manage fatigue). Importantly, participants learn how to develop a formal action plan to motivate and sustain such behavior changes. A full description of the ADT Educational Program can be found in Wibowo et al. (2020); further information is also available at the program website, www.LifeOnADT.com [12].

### 2.3. Measures

*Socio-Demographic and Health History Questionnaire:* A socio-demographic and health history questionnaire was designed by the study team. This questionnaire included items about age, ethnicity, relationship status, education level, employment status, socioeconomic status (household income), PCa treatment history, previous PCa treatments, and health co-morbidities.

*ADT Management Strategies Inventory (ADT-MSI):* Based on the formatting of the Expanded Prostate Cancer Index Composite (EPIC) [23], the ADT-MSI was designed by the study team and previously assessed via confirmatory factor analysis (see Wibowo et al., 2020) [12]. The ADT-MSI is divided into two parts to evaluate: (I) use of side effect strategies, and (II) frequency, bother, and self-efficacy to manage specific ADT side effects. While the full ADT-MSI was used for the in-person classes, and data on side effect management was reported in Wibowo et al. (2020), an abbreviated version involving just Part II of the MSI was used for online classes. For Part II of the MSI, the occurrence of side effects is rated dichotomously as “yes” (experienced in the past month) or “no”. Side effect bother is assessed using a 5-point Likert-scale question with responses ranging from “0—no problem” to “4—big problem”. Self-efficacy to manage side effects is assessed with an 11-point Likert-scale with responses ranging from “0—not confident at all” to “10—very confident”.

### 2.4. Procedures

Postcard advertisements were distributed to patients via new-patient orientation packages, alongside ADT prescriptions, and directly from providers to patients. In-service style presentations were offered to specialist pharmacy team members, physicians, and nurses in urology, as well as those in medical and radiation oncology. Information about the program was shared with local PCa support groups. Ethics approval was obtained at each site.

Patients who were interested in attending an ADT class first spoke with a project assistant by phone or via email to discuss the ADT program and to register for an upcoming class. During this initial call, the Project Assistant introduced the study and inquired as to the patient’s willingness to participate in the formal program evaluation. Participation in the program evaluation was optional. Patients were deemed ineligible if they had metastatic disease and high symptom burden, if they were also undergoing chemotherapy, or if they were planning to be on ADT for less than 6 months. Those who were eligible and interested in participating were sent via email an online consent form and baseline questionnaire package. Of the 360 patients who attended an online class during the study period (January 2019–August 2021), 284 initially agreed to participate in the program evaluation study.

Following informed consent, baseline questionnaires were administered prior to class attendance; follow-up questionnaires were administered approximately 3 months later. Those who attended in-person classes were invited to complete questionnaires in hardcopy or online via REDCap survey software (Version 10.6.28). REDCap was used to administer questionnaires to online class participants. Compensation was provided to participants to cover parking costs for those who attended in person. For a full description of procedures used for the in-person classes, see Wibowo et al. (2020) [12].

### 2.5. Data Analysis

Side effect-related outcomes: An overall score for each of the five side effect categories (i.e., for body feminization, physical changes, psychological changes, sexual changes, medical risks) was calculated for all side effect-related outcomes (i.e., *occurrence*, *bother*, *self-efficacy*). For side effect *occurrence*, participants were scored dichotomously, with “0” if they endorsed none of the side effects within a category and “1” if they endorsed at least one side effect within each category. In terms of *self-efficacy* to manage side effects, a final score was calculated as the average of scores within each side effect category (e.g., a final score for self-efficacy to manage sexual side effects was calculated as the average of self-efficacy to manage erectile dysfunction and self-efficacy to manage loss of sexual desire). The same procedure was followed for the calculation of final scores for the degree of side effect *bother*. Consistent with previously published findings [12], the fifth side effect category, ‘medical risks,’ was not included in MANOVA analyses. As described in Wibowo et al. (2020), the ‘medical risks’ category was deemed to be distinct from the other four categories. Patients were simply asked if they considered themselves to be at risk for certain medical conditions and not if they were currently experiencing them.

MANOVA: Differences over time and between groups: Three 2 (pre-post) × 2 (between-group: in-person versus online) mixed MANOVAs were conducted to assess changes in: (1) side effect occurrence, (2) side effect bother, and (3) self-efficacy to manage side effects. For each MANOVA, the same four dependent variables were used. These were based on previously established categories of side effects: physical, psychological, sexual, and body feminization (Wibowo et al., 2020). According to previous studies in which patient experiences of ADT side effects were examined [22], age was included in the models as a key predictor variable.

## 3. Results

### 3.1. Characteristics of Participants

Ninety-four participants from in-person classes and 137 participants from online classes completed baseline and follow-up questionnaires. The average age of participants was nearly identical across participants from in-person (M = 68.39 years, SD = 7.68, range = 48–85) and online (M = 68.72, SD = 6.59, range = 49–84) class formats. Similar relationship status was observed across both class formats, where 82% (*n* = 77) of in-person and 88% (*n* = 121) of online class participants were in a relationship. The ethnicity of participants was homogenous, with 84% (*n* = 79) of in-person participants and 91% (*n* = 124) of online class participants self-identifying as White. In terms of ADT history, 85% (*n* = 80) of participants from the in-person classes and 77% (*n* = 106) from the online classes endorsed having already started ADT. Further demographic and health details are available in Table 1, while Table 2 contains information about where the participants learned about the ADT program.

### 3.2. Baseline and Follow-Up Reports

Additionally, a series of MANOVAs were conducted for side effect occurrence, side effect bother, and side effect self-efficacy. A full presentation of these results can be found in Table 3. Summaries of baseline and follow-up side effect occurrence, side effect bother and side effect self-efficacy are reported in Table 4.

### 3.3. Side Effect Occurrence

MANOVA results for differences in side effect occurrence between in-person and online class formats indicate a significant multivariate effect (Λ = 0.899, *F*(1, 192) = 5.500, *p* < 0.001, Table 3). Statistically significant differences in average ratings of side effect occurrence were observed, with higher scores reported among those who attended in person two of the four side effect categories: physical (M = 0.988 vs. M = 0.871 for in-person vs. online, respectively) and sexual (M = 0.914 vs. 0.875 for in-person vs. online, respectively). In terms of overall pre-class to post-class differences in occurrence scores, there was no significant difference (*p* = 0.091). However, when looking at specific side effect categories, the occurrence of body feminization side effects differed from pre-class to post-class (*p* = 0.022), with the average score increasing from pre-class (M = 0.556) to post-class follow-up (M = 0.878) across both class formats. There were no significant changes over time in the other side effect categories.

**Figure 1:**
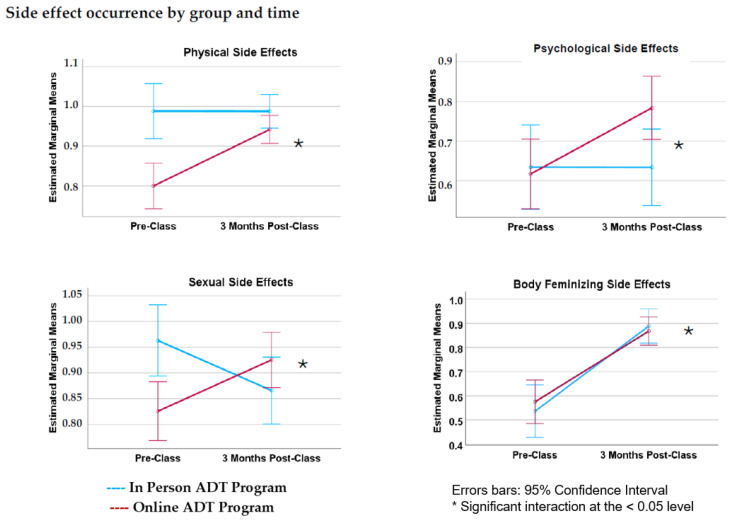
Graphs of changes in side effect occurrence for participants before and after attending the ADT class. Overall, participants in the in-person class rated the occurrence of *physical* and *sexual* side effects higher at baseline than did those who attended the online class. Average ratings of the occurrence of *body feminizing* increased significantly before and after the class for participants in both in-person and online groups. A significant interaction between time and class format was found for *physical*, *sexual*, and *psychological* side effects. *Physical*, *sexual*, and *psychological* side effects tended to increase over time for those who attended the online class, whereas side effect occurrence generally stayed constant or decreased over time for participants in the in-person class.

Furthermore, there was a significant interaction between time point (pre- versus post-class) and class format (in-person versus online; Figure 1). Overall pre-post changes in side effect occurrence therefore differed according to class format (Λ = 0.897, *F*(1, 192) = 5.648, *p* < 0.001). This interaction between time and class format was significant for 3 of the 4 side effect categories: physical (*p* < 0.001, η_p_^2^ = 0.051), sexual (*p* < 0.001), and emotional (*p* = 0.017). Age was entered as a covariate but did not result in significant main effects; similarly, there was no significant interaction between time point and age. Summaries of baseline and follow-up side effect occurrence scores are provided in Table 4.

### 3.4. Side Effect Bother

**Figure 2:**
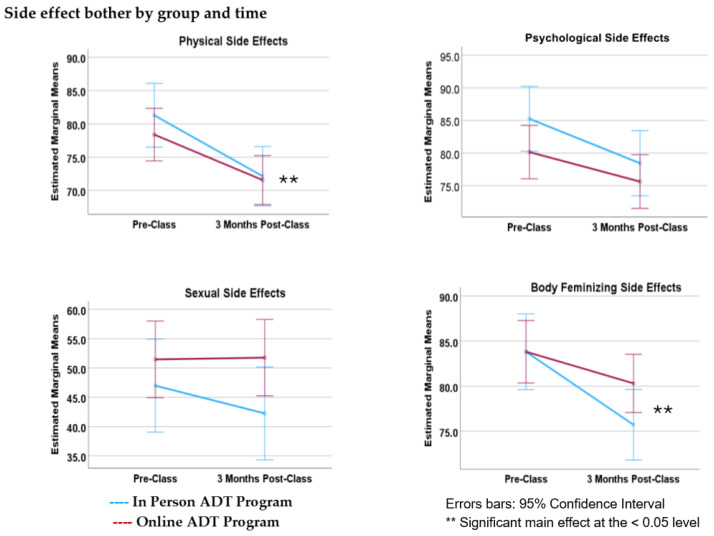
Results of a mixed MANOVA assessing side effect-related bother according to class format and time, as in the above figure. There was a significant multivariate effect. Average self-reported ratings of bother severity decreased for all side effect categories, but statistically significant reductions in bother were observed for only *physical* and *body feminizing* effects. Overall side effect bother scores improved for both in-person and online class participants, with no significant differences according to class format.

MANOVA results for overall pre-post changes in side effect bother indicate a significant multivariate effect (Λ = 0.920; *F*(1, 211) = 4.572, *p* = 0.001, Table 3). According to results of post hoc comparisons, decreases in bother scores were observed for all categories; however, statistically significant decreases were observed for only two of the four side effect categories; i.e., physical side effects (*F*(1, 211) = 12.255, *p* < 0.001) and body feminization effects (*F*(1, 211) = 11.749, *p* < 0.001) (Figure 2). The overall reduction in bother scores from pre-class to post-class did not significantly differ according to class format (Λ = 0.980; *F*(1, 211) = 1.041, *p* = 0.387). Age was entered into the model as a covariate; there were no significant main effects. There was, however, a significant interaction between age and time point for two of the four side effect categories: physical side effects (*F*(1, 211) = 8.028, *p* = 0.005) and body feminization effects (*F*(1, 211) = 8.597, *p* = 0.004). Summaries of baseline and follow-up side effect bother scores are provided in Table 4.

### 3.5. Side Effect Self-Efficacy

There was a statistically significant overall difference in self-efficacy scores between online versus in-person classes. MANOVA results for between-subjects differences in side effect self-efficacy indicate a significant multivariate effect (Λ = 0.871; *F*(1, 92) = 7.059, *p* < 0.001, Table 3). There was, however, no significant difference between overall pre-class and post-class self-efficacy scores (*p* = 0.267). Although not statistically significant, the average self-efficacy rating for participants in both the in-person and online classes increased from T1 to T2 for all side effect categories.

**Figure 3:**
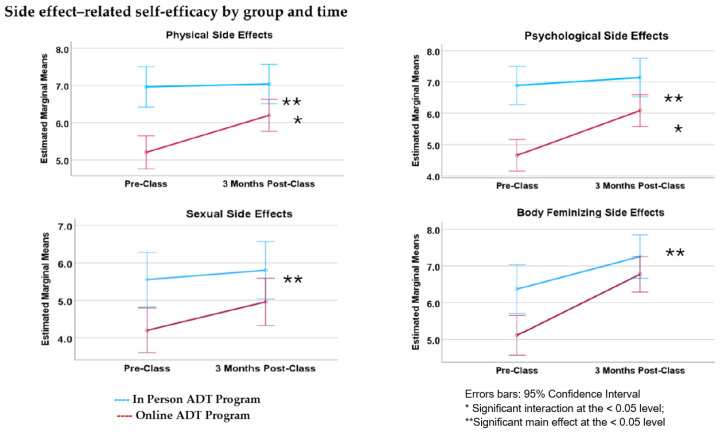
Results of a mixed MANOVA assessing participants’ self-reported sense of self-efficacy to manage side effects, according to class format and time. There was a significant multivariate effect (*p* < 0.001). A statistically significant difference in participants’ overall self-efficacy scores between the in-person and the online class was found. Those who attended in-person classes reported higher overall side effect-related self-efficacy at both baseline and follow-up compared to those who attended online. Further, there was a statistically significant interaction between time and class format (*p* = 0.049) for *physical* and *psychological* side effects. Although not statistically significant, the average self-efficacy rating for participants in both in-person and online classes increased from pre-class to 3 months post-class for all side effect categories.

The interaction between time point and class format was statistically significant: Λ = 0.951, *F*(1, 192) = 2.434, *p* = 0.049 (Figure 3). According to post hoc comparisons, the interaction was significant for two side effect categories: physical (*p* = 0.011) and emotional (*p* = 0.003). Age was entered as a covariate but did not result in significant main effects. There was no significant interaction between age and time point for side effect self-efficacy scores. Summaries of baseline and follow-up side effect self-efficacy scores are provided in Table 4.

## 4. Discussion

Androgen Deprivation Therapy, an effective treatment for PCa, comes with a multitude of side effects that can diminish PCa patients’ health-related quality of life and overall well-being [1,6,7]. The need for programs to support PCa patients’ self-management of side effects is well established [4,5,24,25]. In recent years, there has been a shift toward offering psychosocial interventions online rather than in-person, thereby increasing accessibility for patients outside of the catchment area of major cancer centers [26,27]. However, online supportive care interventions warrant rigorous evaluation to ensure their effectiveness relative to face-to-face options [26].

Here we assessed the relative effectiveness of the ADT Educational Program in an online versus in-person format. Socio-demographic characteristics and ADT history were consistent for participants in both class formats. Additionally, the majority of participants in both class formats had already started ADT prior to attending the class (85% of in-person, 77% of online participants).

Post-class changes in participants’ self-reported side effect occurrence, severity of bother related to side-effect experiences, and sense of self-efficacy to manage side effects were examined. The online format was found to be non-inferior to the in-person class format in alleviating bother associated with ADT side effects. Participation in both online and in-person classes led to significant improvements over time in the self-reported severity of bother from physical and body-feminizing side effects. These findings of reduced bother from treatment side effects are consistent with cancer research comparing the effectiveness of face-to-face versus online groups involving a range of psychosocial interventions; e.g., cognitive behavior therapy, nutritional counseling, exercise programs [26]. In a recent study by Lleras de Frutos et al. (2020), for example, no significant differences were found between an in-person versus online group positive psychotherapy program in terms of sustained improvements in emotional distress and post-traumatic growth [28].

### 4.1. Side Effect Occurrence

Across all categories, side effects reported by online and in-person class participants were similar overall. When looking at specific categories, however, there were significantly fewer physical (i.e., fatigue, genital shrinkage, weight gain, muscle loss) and sexual (i.e., erectile dysfunction, loss of sexual desire) side effects reported in the online group at both baseline and at three months post-class. This may be due to slight differences between participants in each group in terms of the length of time they were on ADT before they took the class. It was expected that with increased time on ADT, individuals would experience more side effects; therefore, between pre- and post-class, side effects would expectedly increase. While a significant increase in body feminizing side effects (i.e., hot flashes, breast tenderness, breast enlargement) was observed for participants in both class formats, significant changes over time were not observed for the other categories.

For several side effect categories, physical, sexual, and emotional, a significant interaction between time and class format was observed. Side effect occurrence generally increased between pre- and post-class for online class participants, but remained stable or decreased over time for in-person class participants (Figure 1). Given that online classes commenced more recently, it is possible that participants in online classes were taking newer and/or different combinations of ADT drugs. Indeed, ADT treatment intensified with the addition of ARPIs, more commonly prescribed in recent years [8]. With more ADT agents used in combination, more side effects are experienced [29]. In contrast to findings from previous research, which demonstrated an inverse relationship between side effects and age (i.e., more side effects among younger men) [22], our results indicated no significant effect of age.

### 4.2. Side Effect Bother

Side effect bother decreased from baseline to three-month follow-up for participants in both class formats (Figure 2). This finding contrasts with previous work by Wibowo et al. (2020) in which bother increased from pre-class to post-class for participants in the in-person classes [12]. Results of post hoc analyses revealed that decreases in bother were statistically significant for physical and body feminizing side-effect categories only. Although bother associated with emotional and sexual side effects also decreased, the effect was not statistically significant. Notably, age as a covariate appeared to contribute to changes in bother scores over time for these same two categories.

### 4.3. Side Effect Self-Efficacy

Participants’ sense of self-efficacy to manage ADT side effects differed significantly for participants in the online versus in-person classes, with higher overall self-efficacy scores for all side effect categories observed for in-person class participants. In the in-person group, self-efficacy scores were generally higher at baseline and remained higher at follow-up (Figure 3). Additionally, MANOVA results reveal a significant interaction between time point and class format only for physical and emotional side effects. In other words, the effect of time on self-efficacy scores for physical and emotional side effects differed according to class format (i.e., in-person versus online). Self-efficacy scores among online class participants were lower at baseline and increased more steeply than did the scores of in-person participants (Figure 3). It is possible that experiences of in-person versus online classes affected self-efficacy differently, perhaps due to a level of comfort associated with joining an online class from one’s home [30].

Previous studies assessing impacts of online interventions on self-efficacy among oncology populations have mixed findings [17,31,32,33]. One study aiming to improve self-efficacy following an online health and self-management intervention for 325 breast and prostate cancer patients documented declines in both self-efficacy and health-related quality of life among control group participants [31]. While improvements in self-efficacy in the current study were not statistically significant, we are pleased that they contrast with studies that show declines in self-efficacy without intervention. Given the importance of self-efficacy in promoting good quality of life and reducing the burden of disease, it is worth exploring this effect further in future studies. Indeed, this has been suggested by other authors, who report an ongoing need to improve self-efficacy for PCa patients in terms of managing treatment side effects [24,34].

### 4.4. Limitations

The study sample was comprised predominantly of White, heterosexual, educated men. Generalizability of findings may be limited. Future research might endeavor to increase recruitment from more diverse groups (e.g., minority and marginalized populations) [35]. Findings pertain to patients with access to well-resourced cancer care. The ADT Educational Program would be less applicable to parts of the world where there is little or no screening for prostate cancer and patients are largely diagnosed with symptomatic metastatic disease…or where androgen suppression medications are less available. This study also did not include a control group, so we were not able to analyze data from patients on ADT who did not participate in the ADT program. As such, we cannot determine if changes in patients’ experiences of side effects are specifically associated with participation in the program, or if these changes might have occurred otherwise with the passage of time. Incorporating randomization into a waitlist control group may help elucidate these effects; however, use of an active treatment control group would be ethically necessary.

There were a number of variables that could not be controlled, including the use of several different facilitators and different teaching sites across in-person and online formats. Additionally, the material covered in the class and the ADT book has evolved over time, in accordance with an expansion of the drugs used for androgen suppression. Patients who participated in the program more recently (i.e., those who attended online) had access to newer editions of the book and updated class content. Similarly, patients who attended more recently were more likely to be on ADT treatments involving newer drugs used in combination (e.g., doublet and triplet therapies). This may be particularly pertinent as some of these treatment regimens are associated with more intense side effects.

## 5. Conclusions

Participating in the ADT Educational Program, whether in the in-person or online format, is associated with significant improvements in the severity of ADT side-effect-related bother. Importantly, bother decreased significantly among both in-person and online class participants, suggesting non-inferiority of the online class delivery. As expected, the occurrence of body-feminizing side effects increased from pre-class to post-class, likely due to the recent initiation of ADT around the time of participating in the program. However, the degree of improvement in patients’ self-reported self-efficacy to manage side effects was not statistically significant.

Our findings provide further evidence that a low-intensity educational intervention, involving one-time attendance in a 1.5 h interactive class plus access to the ADT book, can significantly and positively improve patients’ experience in managing ADT side effects. With its combination of patient education and health behavior-change strategies, the ADT Educational Program offers patients actionable strategies for managing ADT side effects and minimizing side effect bother. The program can reduce the time HCPs need to spend individually educating patients about ADT side effects. The online program can thus be cost-effective for cancer centers that initiate ADT with a large volume of PCa patients.

Overall, this study provides evidence of the effectiveness of an online version of the ADT Educational Program in terms of helping men manage ADT side effects. Online delivery increases accessibility of the program, thereby helping to reduce regional and socioeconomic disparities in care. The online format increases the program’s reach outside metropolitan areas and makes it accessible to those with limited mobility. Given the extent to which ADT side effects infringe upon cardiovascular, metabolic, and psychosocial well-being, it is crucial to offer patients evidence-based programming, whether in person or online, that helps them manage the adverse effects of ADT.

## Figures and Tables

**Table 1:** Sociodemographic and health characteristics of participants from the in-person (*n* = 94) and online (*n* = 137) versions of the ADT Educational Program.

Variable		In-Person		Online	
		*n*	%	*n*	%
Province/Territory (In-Person: *n* = 94; Online: *n* = 137)					
	Alberta	22	23.4	51	37.2
	British Columbia	46	48.9	35	25.5
	Ontario	18	19.1	31	22.6
	Atlantic Provinces	8	8.5	9	6.5
	Saskatchewan	-	-	5	3.6
	Quebec	-	-	2	1.5
	Manitoba	-	-	2	1.5
	Northwest Territories	-	-	1	0.7
	Yukon	-	-	1	0.7
Ethnicity (In-Person: *n* = 93; Online: *n* = 137)					
	White/Caucasian	79	84.0	124	90.5
	Asian/Pacific Islander	7	7.4	3	2.2
	Black/African-Canadian	4	4.3	2	1.5
	First Nations/Aboriginal/Native Canadian	0	0	1	0.7
	Latino/Hispanic/Mexican-Canadian	0	0	1	0.7
	Middle-Eastern/Arab/Indian	2	2.1	1	0.7
	Other	1	1.1	-	-
	Missing	1	1.1	-	-
Relationship (In-Person: *n* = 94; Online: *n* = 133)					
	Yes	77	81.9	121	88.3
	No	17	18.1	12	8.8
	Missing	-	-	4	2.9
Marital Status (In-Person: *n* = 93; Online: *n* = 132)					
	Married/Civil Union	62	66.0	104	75.9
	Divorced/Separated	20	21.2	18	13.1
	Never Married	6	6.4	8	5.8
	Widowed	5	5.3	2	1.5
	Missing	1	1.1	5	3.6
Partner’s Gender (In-Person: *n* = 77; Online: *n* = 121) ^2^					
	Female	75	97.4	113	93.4
	Male	2	2.6	7	5.8
Education (In-Person: *n* = 94; Online: *n* = 133)					
	Graduate or professional degree	44	46.8	61	44.5
	College graduate	13	13.8	33	24.1
	Some college	14	14.9	20	14.6
	High school or technical school graduate	19	20.2	16	11.7
	Less than high school diploma or technical school	4	4.3	3	2.2
	Missing	-	-	4	2.9
Employment Status (In-Person: *n* = 94; Online: *n* = 133)					
	Retired	65	69.1	90	65.7
	Part-time	6	6.4	21	15.3
	Full-time	20	21.3	17	12.4
	Looking for work	3	3.2	5	3.6
	Missing	-	-	4	2.9
Annual Household Income (In-Person: *n* = 87; Online: *n* = 127)					
	CAD 30,001–CAD 100,000	48	51.1	77	56.2
	More than CAD 100,000	28	29.8	38	27.7
	Less than CAD 30,000	11	11.7	12	8.8
	Missing	7	7.4	10	7.3
PCa Treatment Type					
	ADT injections (Yes)	78	83.0	108	78.8
	EBRT (Yes)	27	28.7	54	39.4
	Radical Prostatectomy (Yes)	42	44.7	47	34.3
	ADT pills (Yes)	38	40.4	47	34.4
	Active Surveillance (Yes)	18	19.1	12	8.8
	Brachytherapy (Yes)	5	5.3	9	6.5
	Cryotherapy (Yes)	1	1.1	1	0.7
	Orchiectomy (Yes)	0	0	0	0
	Other (Yes) ^1^	-	-	13	9.5
ADT Started Prior to Class (In-Person: *n* = 94; Online: *n* = 136)					
	Yes	80	85.1	97	70.8
	No	14	14.9	39	28.5
	Missing	-	-	1	0.7
		M (SD)	Range	M (SD)	Range
Age (Years)		68.39 (7.68)	48–85	68.72 (6.59)	49–84
Relationship Duration (years) (In-Person: *n* = 77; Online: *n* = 121)		33.99 (16.87)	0.5–62	36.00 (15.46)	0.6–59
Duration (days) between baseline and T2 follow-up (In-Person: *n* = 99, Online: *n* = 133)		84.05 (27.23)	52.00–219.00	84.42 (16.05)	68.53–188.32
Anticipated duration (number of weeks) between registration form and start of ADT (Online: *n* = 37)		-	-	7.65 (11.90)	0.00–52.00

^1^ Other = “began 31 July 2019”; “cessation of androgen replacement therapy”; “chemotherapy”; “complementary natural products”; “Just started 5 August”; “naturopathic treatments”; “not yet”; “palliative radiotherapy”; “radiation and chemotherapy”; “started 2 September 2020”; “Metformin (to help with radiation, not for diabetes)”; “TURP”; “TURP procedure”. ^2^ Among online class participants, 120 of the 121 partnered participants provided a response regarding the gender of their partner.

**Table 2:** Source of referral to online ADT Educational Program, as per participants’ self-report via program registration form.

Referral Source	Frequency	Percent
Clinic Nurse/Physician	40	17.3
Support Group/Webinar	35	15.2
Website/Internet Search	16	6.9
Patient/Peer/Friend	12	5.2
Poster/Pamphlet	10	4.3
Program Facilitator	6	2.6
Psychosocial Oncology Clinician	3	1.3
Pharmacy	3	1.3
ADT Book	3	1.3
Prostate Cancer Centre	3	1.3
Organization Newsletter	1	0.4
Other	1	0.4
Missing	98	42.4
Total	231	100.0

**Table 3:** Comparison of in-person and online class participants’ scores (between subjects) from pre-class to post-class (within subjects) on side effect variables (MANOVA).

Outcome			F	df	*p* Value	Partial Eta Squared
Side effect occurrence scores						
	In-person vs. online		**5.500**	**1, 199**	**<0.001**	**0.101**
		Body Feminization	0.035	1, 199	0.852	0.000
		Physical	**14.376**	**1, 199**	**<0.001**	**0.067**
		Emotional	1.346	1, 199	0.247	0.007
		Sexual	1.479	1, 199	0.225	0.007
	Pre-class vs. post-class		2.032	1, 199	0.091	0.040
		Body Feminization	**5.330**	**1, 199**	**0.022**	**0.026**
		Physical	1.389	1, 199	0.240	0.007
		Emotional	0.652	1, 199	0.421	0.003
		Sexual	0.133	1, 199	0.716	0.001
Side effect bother						
	In-person vs. online		2.216	1, 212	0.068	0.041
		Body Feminization	0.914	1, 212	0.340	0.004
		Physical	0.387	1, 212	0.535	0.002
		Emotional	1.808	1, 212	0.180	0.008
		Sexual	2.358	1, 212	0.126	0.011
	Pre-class vs. post-class		**4.572**	**1, 212**	**0.001**	**0.080**
		Body Feminization	**11.749**	**1, 212**	**<0.001**	**0.053**
		Physical	**12.255**	**1, 212**	**<0.001**	**0.055**
		Emotional	0.940	1, 212	0.333	0.004
		Sexual	1.080	1, 212	0.300	0.005
Side effect self-efficacy						
	In-person vs. online		**7.059**	**1, 193**	**<0.001**	**0.129**
		Body Feminization	**6.238**	**1, 193**	**0.013**	**0.031**
		Physical	**18.585**	**1, 193**	**<0.001**	**0.088**
		Emotional	**21.703**	**1, 193**	**<0.001**	**0.101**
		Sexual	**6.349**	**1, 193**	**0.013**	**0.032**
	Pre-class vs. post-class		1.313	1, 193	0.267	0.027
		Body Feminization	2.157	1, 193	0.144	0.011
		Physical	0.030	1, 193	0.862	0.000
		Emotional	0.743	1, 193	0.390	0.004
		Sexual	0.419	1, 193	0.518	0.002

Significant results (*p* < 0.05) are bolded.

**Table 4:** Baseline and follow-up (T2) self-reported side effect occurrence, bother (how much of a problem), and self-efficacy for in-person and online class participants.

	In Person						Online					
	T1			T2			T1			T2		
	Occurrence—Yes	Bother *	Self-Efficacy	Occurrence—Yes	Bother *	Self-Efficacy	Occurrence—Yes	Bother *	Self-Efficacy	Occurrence—Yes	Bother *	Self-Efficacy
Side Effect	*N* (%)	M (SD)	M (SD)	*N* (%)	M (SD)	M (SD)	*N* (%)	M (SD)	M (SD)	*N* (%)	M (SD)	M (SD)
Erectile difficulties	75 (79.8)	40.73 (41.52)	5.27 (2.77)	71 (75.5)	38.33 (43.09)	5.74 (3.19)	101 (73.7)	46.76 (40.12)	4.07 (3.80)	120 (87.6)	51.53 (38.45)	4.96 (3.79)
Loss of libido	56 (59.6)	52.78 (38.84)	5.67 (2.77)	31 (33.0)	47.22 (41.47)	5.78 (3.20)	87 (63.5)	54.04 (39.96)	4.20 (3.68)	115 (83.9)	52.07 (37.69)	4.85 (3.77)
Fatigue	78 (83)	64.89 (35.42)	6.42 (2.28)	71 (75.5)	50.00 (33.54)	6.86 (2.20)	84 (61.3)	58.71 (37.70)	4.82 (3.31)	100 (73.0)	50.95 (33.61)	5.80 (3.20)
Change in genital size	31 (33)	82.88 (29.17)	6.36 (2.45)	51 (54.3)	75.27 (32.38)	6.61 (2.69)	49 35.8)	83.40 (29.19)	4.35 (3.75)	86 (62.8)	77.07 (29.68)	5.32 (3.83)
Weight gain	26 (27.7)	80.00 (29.56)	7.61 (2.05)	43 (45.7)	72.22 (32.98)	7.61 (1.90)	38 (27.7)	81.30 (30.44)	6.08 (3.01)	60 (43.8)	71.80 (33.06)	6.94 (2.76)
Muscle loss	24 (25.5)	84.51 (28.19)	7.17 (2.16)	45 (47.9)	71.70 (30.32)	7.39 (1.88)	41 (29.9)	77.36 (33.69)	5.45 (3.18)	65 (47.4)	66.03 (35.23)	6.50 (3.03)
Relationship strain	20 (21.3)	80.75 (28.45)	6.83 (2.45)	25 (26.6)	82.95 (25.86)	7.29 (2.37)	23 (16.8)	81.68 (30.04)	5.84 (3.79)	30 (21.9)	80.45 (29.89)	6.76 (3.42)
Loss of body hair	13 (13.8)	93.01 (19.63)	-	27 (28.7)	93.68 (16.07)	-	22 (16.1)	93.55 (18.89)	-	42 (30.7)	90.79 (21.64)	-
Breast enlargement	13 (13.8)	91.13 (20.74)	5.88 (2.78)	25 (26.6)	87.09 (23.97)	7.13 (2.33)	26 (19)	91.15 (20.38)	5.02 (3.88)	32 (23.4)	89.77 (21.39)	6.62 (3.51)
Hot flashes	52 (55.3)	65.69 (37.02)	6.82 (2.45)	80 (85.1)	49.45 (31.40)	7.53 (2.23)	71 (51.8)	68.18 (36.82)	5.39 (3.46)	106 (77.4)	56.53 (32.72)	6.65 (3.09)
Emotional expression	33 (35.1)	84.41 (21.47)	6.91 (2.11)	32 (34.0)	76.37 (27.22)	7.24 (2.11)	56 (40.9)	78.85 (29.61)	4.88 (3.30)	72 (52.6)	73.68 (28.92)	6.16 (3.31)
Depression	32 (34)	83.97 (26.62)	6.91 (2.08)	38 (40.4)	79.67 (28.60)	7.19 (2.19)	28 (20.4)	81.54 (29.79)	4.89 (3.41)	51 (37.2)	77.84 (29.08)	6.40 (3.26)
Cardiovascular disease **	-	90.76 (23.65)	6.86 (2.15)	-	87.36 (26.10)	7.36 (1.97)	-	90.96 (24.51)	4.98 (3.71)	-	88.95 (24.79)	6.49 (3.24)
Diabetes **	-	92.93 (21.07)	6.79 (2.25)	-	90.45 (23.08)	7.32 (1.97)	-	89.92 (25.49)	5.25 (3.61)	-	90.96 (21.11)	6.79 (3.24)
Breast Tenderness	7 (7.4)	94.02 (16.31)	6.18 (2.61)	9 (9.6)	93.24 (16.54)	7.20 (2.32)	10 (7.3)	94.19 (16.09)	5.41 (3.92)	13 (9.5)	94.66 (16.70)	7.04 (3.45)
Memory	38 (40.4)	83.97 (25.30)	6.88 (1.96)	43 (45.7)	78.02 (27.09)	7.16 (2.01)	52 (38)	81.15 (27.33)	4.25 (3.47)	72 (52.6)	75.75 (27.69)	5.74 (3.48)
Bone Density Loss	-	89.24 (25.59)	6.18 (2.48)	-	86.25 (23.83)	6.95 (2.19)	-	89.48 (26.71)	4.95 (3.37)	-	85.74 (25.04)	6.13 (3.26)

* Higher numbers indicate lower levels of bother severity (0 = big problem; 100 = no problem). ** Participants were not asked to self-report cardiovascular disease and diabetes.

## Data Availability

Data available on request due to restrictions (e.g., privacy, legal or ethical reasons).
